# REST mediates resolution of HIF-dependent gene expression in prolonged hypoxia

**DOI:** 10.1038/srep17851

**Published:** 2015-12-09

**Authors:** Miguel A. S. Cavadas, Marion Mesnieres, Bianca Crifo, Mario C. Manresa, Andrew C. Selfridge, Carsten C. Scholz, Eoin P. Cummins, Alex Cheong, Cormac T. Taylor

**Affiliations:** 1 Systems Biology Ireland, University College Dublin, Dublin 4, Ireland; 2 Conway Institute of Biomolecular and Biomedical Research, School of Medicine and Medical Sciences, University College Dublin, Dublin 4, Ireland; 3 Instituto Gulbenkian de Ciencia, Rua da Quinta Grande, 2780-156 Oeiras, Portugal; 4 Institute of Physiology and Zurich Centre for Integrative Human Physiology, University of Zurich, Zurich, Switzerland; 5 Life and Health Sciences, Aston University, Birmingham, B4 7ET, UK

## Abstract

The hypoxia-inducible factor (HIF) is a key regulator of the cellular response to hypoxia which promotes oxygen delivery and metabolic adaptation to oxygen deprivation. However, the degree and duration of HIF-1α expression in hypoxia must be carefully balanced within cells in order to avoid unwanted side effects associated with excessive activity. The expression of HIF-1α mRNA is suppressed in prolonged hypoxia, suggesting that the control of *HIF1A* gene transcription is tightly regulated by negative feedback mechanisms. Little is known about the resolution of the HIF-1α protein response and the suppression of HIF-1α mRNA in prolonged hypoxia. Here, we demonstrate that the Repressor Element 1-Silencing Transcription factor (REST) binds to the HIF-1α promoter in a hypoxia-dependent manner. Knockdown of REST using RNAi increases the expression of HIF-1α mRNA, protein and transcriptional activity. Furthermore REST knockdown increases glucose consumption and lactate production in a HIF-1α- (but not HIF-2α-) dependent manner. Finally, REST promotes the resolution of HIF-1α protein expression in prolonged hypoxia. In conclusion, we hypothesize that REST represses transcription of HIF-1α in prolonged hypoxia, thus contributing to the resolution of the HIF-1α response.

Hypoxia is a key microenvironmental feature of a range of physiological and pathophysiological conditions including embryonic development, exercise, cancer, ischemia and inflammation^1^. Adaptive transcriptional pathways have evolved to help an organism deal with the metabolic threat posed by hypoxia. The best-described transcriptional adaptive response in cells is mediated by the hypoxia inducible factor (HIF) signalling pathway, which up-regulates genes which restore oxygen and energy homeostasis^2^,^3^,^4^. In normoxia, HIFα is hydroxylated by the prolyl-hydroxylase domain (PHD) family of dioxygenases targeting it for proteosomal degradation^5^. This process is reversed in hypoxia and HIFα is stabilized, dimerizes with HIFβ and binds to hypoxia response elements (HRE) in the regulatory regions of target genes^6^. HIF drives an adaptive response to hypoxia by promoting the expression of genes including those that regulate erythropoiesis, angiogenesis and glycolysis^6^. However in cancer, HIF signalling can be maladaptive and contribute to tumour survival^1^. Because of the potentially deleterious effects of over-activation of the HIF pathway, a resolution mechanism is required to resolve its activity in prolonged hypoxia. In the absence of such a resolving mechanism, deleterious consequences such as pathologic angiogenesis and excessive haematocrit due to chronic HIF stabilization may occur^7^,^8^,^9^.

While several regulators of HIF expression exist, only a few have been shown to be involved in the resolution of the HIF response to hypoxia. PHD2 and PHD3 are, for example, part of an auto-regulatory mechanism, whereby HIF-1α which is stabilized in hypoxia, transcriptionally induces the expression of *EGLN1* and *EGLN3* genes coding for PHD2 and PHD3 proteins respectively^10^,^11^,^12^. The increased expression of the PHD enzymes in turn promotes HIFα hydroxylation, and reduction of its expression in prolonged hypoxia^10^. Less is known about the control of *HIF1A* mRNA stability^11^. Interestingly, while HIF-1α protein is transiently up-regulated in hypoxia, the mRNA is frequently found to be repressed^12^,^13^,^14^,^15^. This transcript attenuation can be conveyed through mRNA destabilization by the protein tristetraprolin in endothelial cells^14^ and by miR155 in intestinal epithelial cells^12^. The HIF-1α antisense transcript (aHIF) which is widely expressed in both adult and foetal tissue^16^ has also been shown to down-regulate HIF-1α mRNA in lymphocytes, non-papillary renal cell carcinoma and lung epithelial cells^13^,^15^. All these different mechanisms converging on the reduction of mRNA expression in hypoxia indicate that this may be an important component of the cellular adaptation to hypoxia, potentially preventing an over activated HIF response that could be detrimental to cells and tissues^12^. Further supporting this protective role of reduced *HIF1A* mRNA expression in hypoxia, high levels of *HIF1A* mRNA have been observed in hepatocellular carcinoma, gastric cancer and prostate cancer and are often associated with poor prognosis^17^,^18^,^19^,^20^. Of note, the suppression of *HIF1A* mRNA expression in prolonged hypoxia may be dependent on cell type as some reports have reported induction of *HIF1A* mRNA in hypoxia^21^,^22^,^23^,^24^,^25^,^26^.

Both HIF-1α protein and *HIF1A* mRNA are overexpressed in cancer, correlating to poor prognosis. While increased HIF-1α protein levels could be caused by increased oncogenic activity from PI3K/Akt/mTOR and MYC, decreased VHL expression and tumour hypoxia^27^,^28^,^29^,^30^, there is no clearly identified transcriptional mechanism for the over-expression of *HIF1A* mRNA in cancer. NFκB^24^,^31^ and NFAT^32^ have been shown to increase *HIF1A* mRNA expression, however their relative roles in regulating *HIF1A* transcription in the cancer context remain to be fully elucidated. The V-SRC oncogene has been shown to increase HIF-1α mRNA, however the mechanism remains poorly understood^27^. Therefore, the regulation of HIF-1α expression is subject to both conserved post-translational mechanisms and context dependent transcriptional and post-transcriptional events which are necessary for physiological oxygen homeostasis but can also be inappropriately activated in cancer to promote tumour progression.

The Repressor Element 1-Silencing Transcription Factor (REST) is a C2H2- or Krüppel-type zinc finger, one of the largest classes of transcription factors in humans^33^. It binds to the 21 base pair Repressor Element 1 (RE1) on the promoter of target genes and inhibits transcription by regulating chromatin structure or by inhibiting the basal transcription machinery^34^. Proteosomal REST degradation is induced during neuronal differentiation, resulting in the promotion of expression of genes which confer a unique neuronal phenotype^34^,^35^. REST also regulates gene expression in non-neuronal cells^36^,^37^,^38^,^39^,^40^.

While the inhibition of PHD enzymes is fundamental for HIF-1α protein stabilization in hypoxia, much less is known about the resolution of the HIF-1α protein response and the suppression of HIF-1 α mRNA in hypoxia. Analysis of the *HIF1A* promoter revealed the presence of several components of the REST co-repressor complex (REST, mSin3A, CoREST and HADAC2). Due to the previous implication of REST in ischemia we decided to investigate the contribution of REST to HIF-1α regulation in hypoxia.

Here we show that REST regulates cell metabolism by counter-regulating HIF-1α dependent transcription, including the regulation of glycolytic genes. This work also sheds light on our understanding of the crosstalk between transcriptional repressors and activators in the cellular response to hypoxia, a key event in a range of physiologic and pathophysiologic processes.

## Results

### HIF-1α protein is transiently stabilized in hypoxia while its mRNA is suppressed

**Figure 1:**
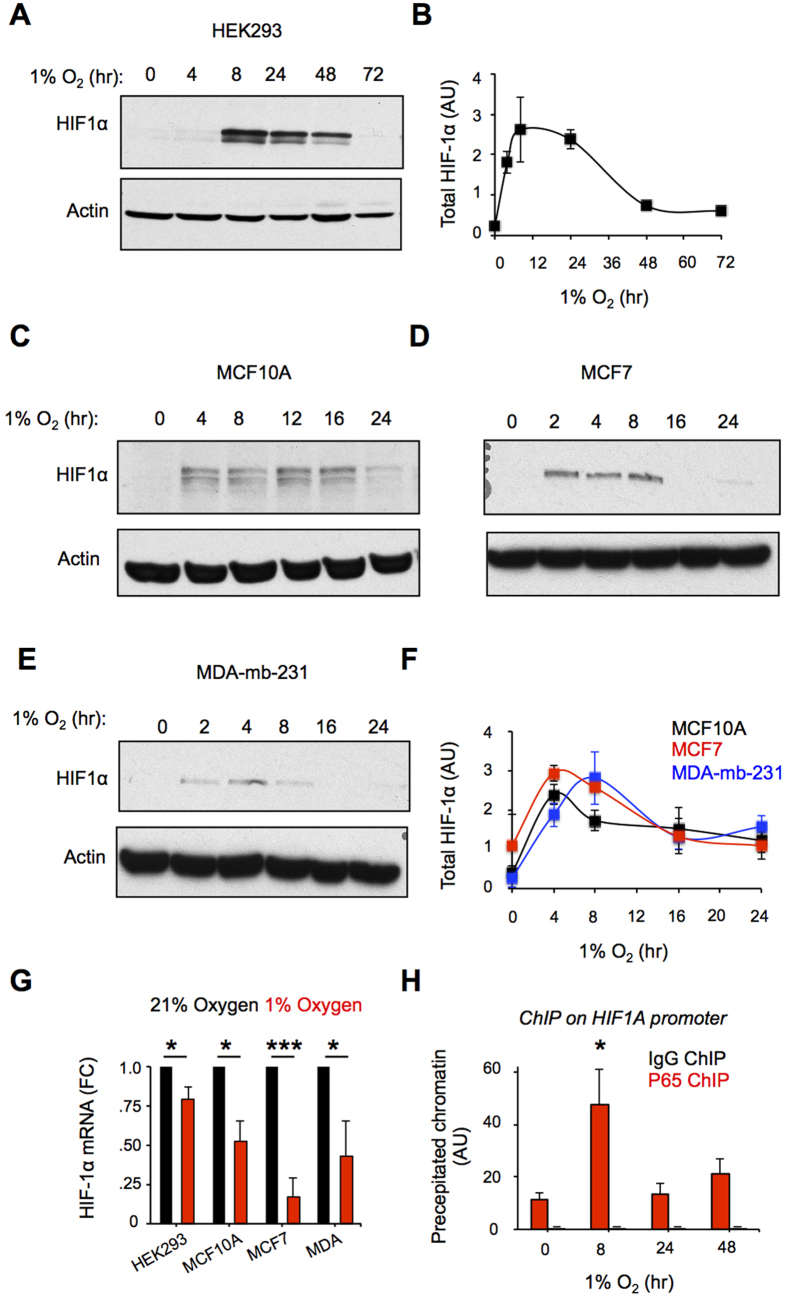
HIF-1α protein is transiently stabilized in hypoxia while its mRNA its suppressed. (**A**) HEK293, (**C**) MCF10A, (**D**) MCF7 and (**E**) MDA-mb-231 cells where exposed to the indicated time point to hypoxia (1% oxygen), protein lysates where prepared and blotted with the indicated antibodies. (**B**–**F**), densitometry of (A) and (**C–E**), respectively. (**G**) Cells where exposed to 8 hours of hypoxia (1% oxygen) or normoxia (21% oxygen), the mRNA was collected and used for qRT-PCR analysis of *HIF1A* mRNA expression. Results are shown as fold change to normoxia. (**H**) ChIP assays coupled to qRT-PCR where performed on HEK293 cells exposed to hypoxia for the indicated time points using p65 and IgG control antibodies, primers covering the NFκB site on the HIF-1α gene where used (see sequence highlighted in blue in Fig. 3A). N = 3-4 independent experiments. Data are represented as mean ± SEM. In (G), *p < 0.05, ***p < 0.001, significant fold change over 21% O_2_. In (**A**), *p < 0.05, significant increase over normoxic p65 ChIP and IgG ChIP.

The HIF-1α response in prolonged hypoxia involves a rapid protein accumulation phase (Fig. 1A–F), followed by a protein resolution phase, where HIF-1α levels decay back to close to normoxic levels (Fig. 1A–F). In the HEK293 cell line, *HIF1A* mRNA expression decreased by only 21% to 32% after 8 hours of exposure to hypoxia (Figs 1G and (3E). In other cell lines including the intestinal epithelial cancer cell line CaCo-2 and in HeLa cells, HIF1A mRNA has been reported to decreased by ~ 50% after 6 to 8 hours of hypoxic exposure (1% oxygen)^12^,^41^. Here, we observed that in normal breast and breast cancer derived cell lines, HIF1A mRNA expression can decrease by 50 to 80% after 8 hours of hypoxic exposure (Fig. 1G). Thus, suppression of *HIF1A* mRNA is common in response to hypoxia.

NFκB has been shown to be responsible for the transient and early *HIF1A* mRNA induction in response to hypoxia, 1% oxygen, in human pulmonary arterial smooth muscle cells^24^. Therefore we decided to test if in HEK293 cells NFκB was recruited to the HIF1A κB element previously shown to bind p50 and p65 in response to hypoxia and activate *HIF1A* transcription^24^. Using chromatin immunoprecipitation assays (ChIP), with a rabbit p65 antibody or rabbit IgG negative control antibody from pre-immune sera, chromatin pull-downs were performed, and qRT-PCR was used to quantify the chromatin containing the HIF1A κB element. This revealed that p65 was transiently recruited to the κB site on the HIF1A promoter in response to hypoxia, being significantly up-regulated in comparison to normoxia and IgG at 8 hours (Fig. 1H). Despite the recruitment of p65 to the κB site on the HIF1A promoter, HIF1A mRNA is still significantly decreased in hypoxia (Figs 1G and 3E), this indicates that counter-regulatory mechanisms must be acting either at the level of regulating mRNA transcription or stability. We next investigated the possibility that a transcriptional repressor was counter-regulating NFκB-dependent activation of *HIF1A* gene transcription (see below).

### REST negatively regulates HIF-1α

**Figure 2:**
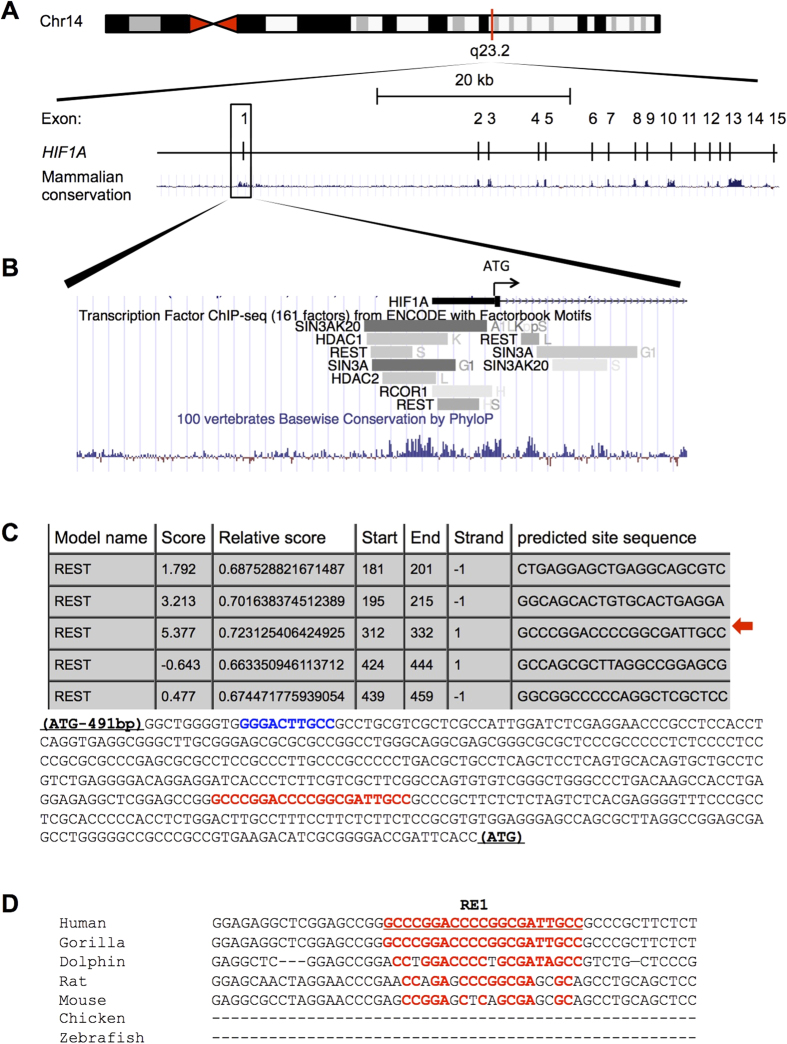
Bioinformatics work flow used to identify the REST binding site (RE1) on the *HIF1A* promoter. **(A)** The *HIF1A* gene with mammalian conservation is depicted in relation to its position on chromosome 14 q23.2 (modified from the UCSC Genome Browser). The human *HIF1A* promoter region -4000 bp upstream to +2000 bp downstream of the translation start site (ATG) was used to screen for the presence of REST-repressor complex components on the ENCODE ChIP-seq datasets. This is a highly conserved region surrounding EXON1. The full list of transcription factors found to be associated on this region of the *HIF1A* gene is described in Fig. S1. **(B)** Close up view on the ChIP-seq hits found for the REST co-repressor complex components: REST, mSin3A, CoREST (RCOR1) and HDAC2 on the *HIF1A* gene promoter. Conservation is depicted by vertical blue bars, bellow the ChIP-seq hits (horizontal grey-scale bars). **(C)** The genomic DNA where REST co-repressor complex ChIP-seq hits was found, was screened using the JASPAR database for the presence of RE1 elements, this revealed a putative RE1 in the -491 bp to 0 bp promoter region (highlighted in red), in close proximity to the previously reported NFκB site on the *HIF1A* gene (highlighted in blue). Red arrow, indicated the highest scoring putative RE1 (Rest binding site) found by JASPAR analysis. **(D)** Conservation of the RE1 site in the *HIF1A* promoter is depicted. See Materials and Methods for more details on the Bioinformatics analysis.

**Figure 3:**
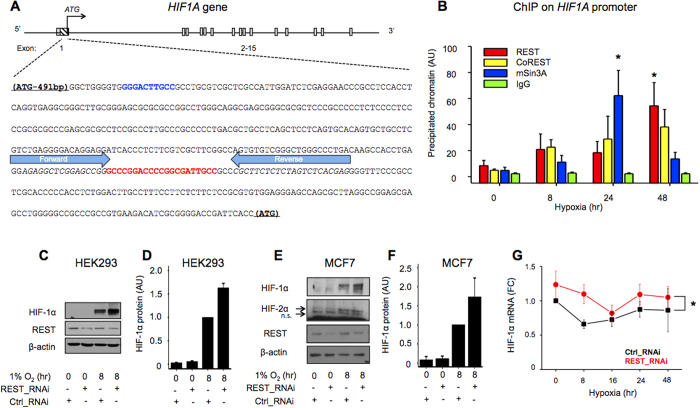
REST negatively regulates Hif-1α. **(A)** Schematic representation of the *HIF1A* genomic locus, grey rectangles indicate approximate Exon position, the white rectangle with diagonal black lines indicates the genomic sequence -491 bp/ATG of the human HIF1A gene. Highlighting is the REST binding site (RE1) in red, and the previously reported NFκB binding site in blue, on the -491 bp/ATG genomic sequence. Blue arrows indicate the primers used to detect REST, COREST, mSin3A and IgG binding to the RE1 site in the ChIP assays. **(B)** ChIP assays on the RE1 site in the *HIF1A* gene promoter using the indicated antibodies in HEK293 cells exposed to the indicated time points to hypoxia (1% oxygen). Precipitated chromatin was quantified by qRT-PCR. **(C–G)** Cells were exposed to hypoxia (1% O_2_) for the indicated time points. REST knockdown was performed using REST specific (REST-RNAi) or control RNAi (**C–G**). Whole cell extracts from HEK293 (**C,D**) and MCF7 (**E,F**) were collected and analysed for the expression of the indicated protein by immunoblotting (**C,E**) and densitometry analysis (**D,F**). HEK293 mRNA was collected and analysed for HIF mRNA expression by qRT-PCR (**G**). F.C. = fold change to 21% O_2_ for (**G**) and control RNAi, 8 hours hypoxia for (**C–F**). Data are represented as mean ± SEM, N = 3–5 throughout. *p < 0.05, significant change over control RNAi.

The protein tristetraprolin^14^, the microRNA miR155, and the lncRNA aHIF^13^,^16^ have all been shown to regulate *HIF1A* mRNA stability, but no example is known of a transcriptional mechanism directly acting on the *HIF1A* promoter. Therefore, we decided to use the ENCODE consortium ChIP-Seq datasets to determine which transcriptional repressors are bound to the human *HIF1A* promoter region, 4000 bp upstream and 2000 bp downstream of the ATG (Fig. 2A,B). This revealed the presence of multiple transcription factors (Fig. S1), among which were members of the REST co-repressor complex, namely REST, mSin3A, RCOR1 (CoREST), and HDAC2 (Fig. 2B)^34^. Due to the previous implication of REST in the response to ischaemia, we decided to test if REST was involved in the repression of *HIF1A* gene transcription. Using the JASPAR database, we looked for putative REST binding sites (RE1) on the *HIF1A* promoter region covered by the ChIP-Seq hits of the REST co-repressor complex family of nuclear factors (Fig. 2C). And identified an RE1 which was evolutionarily conserved within primates, but diverged in other mammals (62 to 81% conserved base pairs in the RE1 site) and was not present in more distant animal species having REST orthologous proteins (Fig. 2D). REST binding to this RE1 site was validated in human HEK293 cells using ChIP assays coupled to qRT-PCR using primers that amplified a 62  bp amplicon containing the RE1 site (Fig. 3A). REST and its co-repressor proteins CoREST and mSin3A were found to be recruited to the HIF-1α gene with similar dynamics (Fig. 3B). Thus, we identified a functional RE1 site in the *HIF1A* promoter that binds REST and its co-repressors CoREST and mSin3A.

To test if REST had a functional effect on *HIF1A* expression, in addition to its binding to the *HIF1A* promoter, REST knockdown using RNAi was used in HEK293 and MCF7 cells. This led to a significant increase in HIF-1α protein (Fig. 3C–F) and mRNA (Fig. 3G), suggesting that REST is a negative transcriptional regulator of HIF-1α expression at least in hypoxia. In HEK293 cells we did not observe up-regulation of HIF-2α in response to hypoxia^42^ and MCF7 cells did not show any change in HIF-2α upon REST knockdown (Fig. 3E,F). We also observed that HEK293 cells overexpressing REST had reduced expression of HIF-1α protein (Figure S2).

Together these findings provide evidence that REST negatively regulates HIF-1α through promoting the the assembly of a REST repressor complex on the *HIF1A* promoter cis-regulatory RE1 element. The data provide insight into the mechanisms underpinning attenuation of *HIF1A* mRNA expression in prolonged hypoxia.

### REST is involved in the resolution of the HIF-1α response

**Figure 4:**
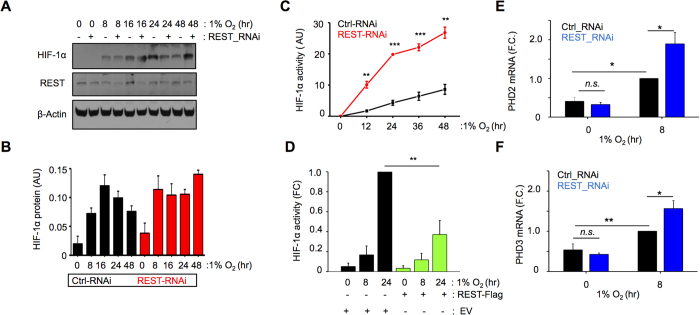
REST is involved in the resolution of the Hif-1α response and in the repression of HIF-1α-dependent transcription. (**A–F**) HEK293 cells were exposed to 21% and 1% oxygen for the indicated time points. REST knockdown was performed using REST-RNAi while REST overexpression was performed with msREST-FLAG construct. (**A**) Whole cell extracts were prepared and immunoblotted as indicated to assess the changes in HIF-1α protein expression under prolonged hypoxia and upon REST knockdown. (**B**) Densitometry of (**A**). (**C**,**D**) HRE-luciferase assays were performed to assess changes in HIF activity upon REST knock-down **(C)** or over-expression **(D)**, using the pHRE-MP-GLuc construct. EV = Empty vector control. FC = Fold change to EV control at 24 hr hypoxia. (**E,F**) mRNA was collected and analysed for the expression of the indicated HIF-1α target genes PHD2 and 3, to assess the changes in endogenous HIF-1α transcriptional activity. FC = Fold change to Ctrl_RNAi in hypoxia. Data are represented as mean ± SEM, N = 3–5 throughout. *p < 0.05, **p < 0.01, ***p < 0.001.

Data presented thus far demonstrates that REST negatively regulates HIF-1α. We next investigated whether REST is involved in the resolution of the HIF-1α-dependent functional response. Typically, *in vitro*, HIF-1α protein accumulates quickly in response to hypoxia, but the response is decreased over prolonged exposure (Fig. 4A, B-black bars). Upon REST knockdown, HIF-1α protein rapidly accumulated in hypoxia, but did not decrease under prolonged hypoxia. Instead the HIF-1α response plateaued (Fig. 4A, B-red bars), indicating that REST may be involved in the resolution of the HIF-1α response. These findings were further confirmed by the increased HIF-1α -reporter activity under prolonged hypoxia when REST is knocked down (Fig. 4C), and decreased HIF-1α reporter activity when REST is overexpressed (Fig. 4D). Endogenous HIF-1α transcriptional activity under REST knock-down was also increased, as accessed by increased mRNA expression of the HIF-1α target genes PHD2 and PHD3 (Fig. 4E,F).

Our data shows that REST plays a role in the suppression of HIF-1α protein expression and transcriptional activity in prolonged hypoxia. Thus there is clear indication for the involvement of REST in the resolution of the HIF-1α response.

### REST regulates glycolytic metabolism in hypoxia

**Figure 5:**
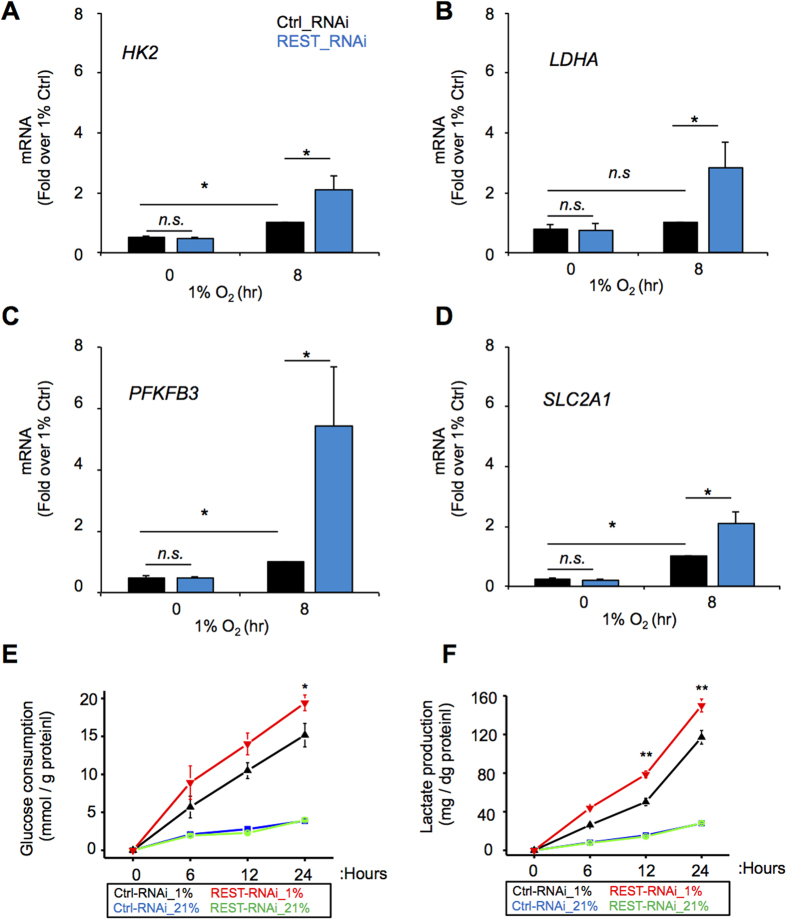
REST loss of function promotes expression of HIF-1α target genes involved in glycolysis, leading to increased glucose consumption and lactate production. (**A–F**) HEK293 cells were exposed to 21% and 1% oxygen for the indicated time points. REST knockdown was performed using REST-RNAi. The mRNA was collected and analysed by qPCR for the expression of the HIF-1α target genes (**A**) HK2, (**B**) LDHA, (**C**) SLC2A1 and (**D**) PFKFB3. (**E**) glucose consumption and (**F**) lactate production assays where performed. Data are represented as mean ± SEM, N = 3–6 throughout. *p < 0.05, **p < 0.01.

Having observed that HIF-1α activity is increased under prolonged hypoxia when REST levels are decreased by RNAi, we next investigated whether this is reflected by altered functional responses to hypoxia. The mRNA expression levels of four well described HIF-1α target genes encoding proteins known to be involved in glucose metabolism (HK2, LDHA, SLC2A1 and PFKFB3) were measured. In hypoxia, the expression of these genes was increased upon loss of REST (Fig. 5A–D). These results suggest that REST loss could affect glucose metabolism by increasing glucose consumption. We measured glucose consumption and lactate production in HEK293 cells and observed that, in hypoxia (1% oxygen), knockdown of REST resulted in enhanced glucose consumption and lactate production (Fig. 5E,F). Thus we show that REST supresses hypoxia-induced glucose consumption and lactate production via repression of HIF-1α. In conclusion, we identify REST as a repressor of gene expression in hypoxia which acts through the repression of HIF-1α-dependent transcription.

## Discussion

The regulation of HIF-1α expression is well documented at the protein level, but much less is known about the control of its mRNA stability^11^. Interestingly, while HIF-1α protein is transiently up-regulated, the mRNA is actually repressed^12^,^13^,^14^,^15^. This transcript attenuation can be conveyed through mRNA destabilization by the protein tristetraprolin in endothelial cells^14^ and by miR-155 in intestinal epithelial cells^12^. The HIF-1α antisense transcript (aHIF), which is widely expressed in both adult and foetal tissue^16^, has also been shown to down-regulate HIF-1α mRNA in lymphocytes, non-papillary renal cell carcinoma and lung epithelial cells^13^,^15^. All these different mechanisms converging to the reduction of mRNA expression in hypoxia indicate that this is an important adaptation to hypoxia, potentially to prevent an over activated HIF response that could be detrimental to cells and tissues^12^. Further supporting this protective role of reduced HIF-1α mRNA expression in prolonged hypoxia, high levels of HIF-1α mRNA have been observed in hepatocellular carcinoma, gastric cancer and prostate cancer and often associated to poor prognosis^17^,^18^,^19^,^20^.

Here, we demonstrate the existence of a transcriptional repressor mechanism acting on the *HIF1A* promoter response to prolonged hypoxia. This data together with the recruitment of p65 to the *HIF1A* promoter (Fig. 1H) indicates that *HIF1A* transcription is under the tight control of both a transcriptional activator and a repressor in response to hypoxia, and de-regulation of this balance might contribute to changes in *HIF1A* mRNA expression. This can be expected to occur in situations where p65 is activated including inflammatory settings^43^ and when REST is down-regulated including cancer^35^. Of interest, other examples exist of genes counter-regulated by NFκB and REST^44^. The neurotransmitter gene *TAC1* is repressed in mesenchymal stem cells (MSC) and their normal lineage differentiated progeny Bone Marrow (BM) stromal cells, by REST and NFκB, but not in retinoic acid differentiated cells used to induce neuronal trans-differentiation of MSC^44^. Of notice, although NFκB activation is observed after 8 hours of hypoxia (Fig. 1H), no induction of HIF1A mRNA is observed in REST knockdown cells (Fig. 3G), indicating REST-independent mechanisms involved in the repression of NFκB -induced transcription (e.g. tristetraprolin, miR-155 and aHIF).

REST knockdown had no effect on HIF-2α protein expression (Fig. 3D). Of notice, miR-155 and aHIF, known regulators of *HIF1A* mRNA also exhibit specificity towards HIF-1α, without an effect on *EPAS1* (gene coding for HIF-2α) mRNA expression^12^,^13^. *EPAS1* mRNA is generally not repressed by hypoxia like *HIF1A* mRNA^12^,^13^, it is therefore not entirely surprising that the mechanisms that lead to *HIF1A* mRNA repression in hypoxia do not affect *EPAS1* mRNA expression. In conclusion, REST regulates HIF-1α, but not HIF-2α expression at least in MCF7 cells.

The tumour suppressor role of REST in non-neuronal cells has been linked to proliferative pathways^35^,^38^. However, the exact mechanisms whereby REST induces these changes remain unknown^35^. In this work we have shown that REST reduces HIF-1α expression and glycolysis in hypoxia, two promising targets for cancer therapy^45^. Thus our molecular biology findings have the potential to be of clinical importance in cancer, and expands the current knowledge about the mechanisms whereby REST might exert its tumour suppressor role. Our data suggest that in the absence of REST, as it occurs in REST-less tumours, there is no repression of *HIF1A* transcription, therefore positive regulators like NFκB are able to freely drive *HIF1A* transcription, leading to increased HIF-1α protein expression in hypoxic tumour microenvironments. Indeed, we observe that the tumour cell line HepG2, which displays HIF1A mRNA induction in hypoxia, has lower REST level than HEK293 (Figure S3). HIF activates several processes that confer an advantage for the development of cancer^46^. Among these processes is glycolysis, which facilitates energetic adaptation to the hypoxic environment^47^.

In addition, glycolysis is also known to be correlated to increased tumour aggressiveness, as it facilitates acidosis, eliminating normal cells surrounding the acidic environment or inducing mutations that will transform these cells, and is able to stimulate *in vitro* invasion and *in vivo* metastases, potentially by inducing extracellular matrix degradation^46^,^47^.

REST is a tumour suppressor in non-neuronal cancers of the breast, colon and lung, therefore increasing the range of pathophysiological settings were our findings might be of importance in the regulation of the HIF-1α response and glycolysis^45^. HIF-1α regulation by oncogenic pathways has been shown to operate mostly by increasing protein expression or decreasing its degradation^45^,^48^. Over-activation of the PI3K/Akt/mTOR pathways leads to increased *HIF1A mRNA* translation and inactivation of VHL can lead to decreased HIF-1α protein degradation^27^,^28^,^29^,^30^,^45^,^49^.

*HIF1A* mRNA is also overexpressed in cancer, correlating to cancer aggressiveness^11^,^17^,^50^. However, there is no clear known mechanism explaining this overexpression^45^. The transcription factors NFκB^24^,^31^ and NFAT^32^ are described to transcriptionally activate *HIF1A* in response to inflammatory stimuli and in activated mast cells, respectively. In many situations the cancer microenvironment can be inflamed, and inflammation can drive cancer development, but the roles of NFκB and NFAT in regulating *HIF1A* in this context remain to be fully elucidated^11^. The V-SRC oncogene has been shown to increase *HIF1A* mRNA, however the mechanism remains poorly understood^27^.

In this work, we have observed that loss of REST by genetic manipulation leads to increased HIF-1α expression, while increased REST levels lead to reduced HIF-1α expression. Thus REST is a tumour suppressor gene that transcriptionally represses *HIF1A*. We hypothesize that loss of the tumour suppressor REST^51^ being the only known transcriptional repressor of *HIF1A* transcription, would allow the positive transcriptional regulators of *HIF1A* to drive its transcription, explaining its over-expression in several cancers. Taken together these observations suggest that our findings may be of clinical importance, as they provide insight into the crosstalk between hypoxia, HIF-1α, glycolysis and REST, all of which play important roles in solid tumours^35^,^52^.

We have shown that in hypoxia REST is recruited to the *HIF1A* promoter, together with the co-repressors CoREST and mSin3A. These co-repressor complexes have associated enzymes with chromatin modifying activity including histone deacetylases, histone methytransferases and demethylases, and methyl-CpG-binding protein 2 (MeCP2)^34^, thus suggesting that REST serves as a platform for the hypoxia induced epigenetic silencing of *HIF1A* expression in hypoxia. This would counteract the recruitment of NFκB to the *HIF1A* promoter, which has been associated to increased transcription in hypoxia^24^. Together these two regulators REST and NFκB may act to fine tune *HIF1A* expression according to environmental clues, and de-regulation of the expression of one of these transcription factors is expected to affect *HIF1A* expression, as it can happen in inflammatory diseases^43^ and REST-less tumours^35^. Of interest MeCP2, a protein often associated with the long term silencing by the REST co-repressor complex, and usually found in the vicinity of the RE1 element^34^,^53^, has been found in the *HIF1A* promoter region, upstream of the ATG, in close proximity to the RE1 we identified on *HIF1A*^54^.

In clear cell renal cell carcinoma (CCRCC) VHL-defective RCC4 and RCC10 cells, binding of HIF-1α/2α to the reverse HRE (rHRE) on the *HIF1A* promoter, leads to H3K9 methylation, H3K4 de-methylation and *HIF1A* repression^55^. Binding of HIF-α subunits to rHREs has been described as a mechanism to repress transcription in hypoxia^56^,^57^. Interestingly, these methylation changes are classical marks of REST-mediated epigenetic silencing^34^. The authors of the previous study speculate on the recruitment, through as yet unknown mechanisms, of complexes carrying histone modifying activities to mediate these epigenetic changes^55^. Of note this rHRE is located up-stream of the ATG, in close proximity to the RE1 we identified. In summary, the *HIF1A* promoter contains epigenetic marks associated with REST (*e.g.* methylated H3K9) or chromatin modifying proteins associated with the REST co-repressor complex (*e.g.* MeCP2), this together with our findings of the recruitment of REST and its co-repressor complexes mSin3A and CoREST to the *HIF1A* promoter suggests a role for REST as an epigenetic regulator of *HIF1A*.

In conclusion, our findings have identified REST as a key repressor of *HIF1A* gene transcription and it’s downstream glycolytic genes, playing a key role in cellular adaptation to hypoxia.

## Materials and Methods

### Cell culture

Human embryonic kidney cells (HEK-293) and human tumorigenic mammary epithelial cells (MCF7 and MDA-MB-231) were cultured in Dulbecco’s modified eagle medium (DMEM, high glucose 4.5 g/L without pyruvate) supplemented with 10% foetal calf serum (FCS) and 100 U/mL penicillin-streptomycin (PS). Human non-tumorigenic mammary epithelial cells (MCF10A) were grown in DMEM/F12 with 5% horse serum, 100 U/mL PS, 2 mM L-Glutamine, 10 μg/mL insulin, 20 ng/mL Epidermal Growth Factor (EGF), 0.5 μg/mL hydrocortisone and 100 ng/ml cholera toxin. Human liver carcinoma HepG2 cells were maintained in minimum essential medium containing 10% FCS, 2 mM L-glutamine, nonessential amino acids, and 100 U/ml PS. All cells were obtained from the American Type Culture Collection (ATCC). All reagents for cell culture were from Gibco (Life Technologies, Calrsbad, CA, USA), unless otherwise stated. Cells were exposed to hypoxia using pre-equilibrated media and maintained in standard normobaric hypoxic conditions (1% O_2_, 5% CO_2_ and 94% N_2_) in a hypoxia chamber (Coy Laboratories, Grass Lake, Michigan, USA). Normoxic controls were maintained at atmospheric O_2_ levels (21% O_2_, 5% CO_2_ and 74% N_2_) in a tissue culture incubator.

### Gaussia luciferase assay, transient and stable transfections

Gaussia luciferase assays were performed as previously described^58^. Briefly, at the selected time points, 10 μL of media was collected from the supernatant and stored at –20 °C. Gaussia luciferase activity was measured using the Biolux Gaussia luciferase Flex Assay kit (NEB) in a plate reader (Synergy HT, Biotek) and normalized to the luciferase activity of the secreted cypridina luciferase under the control of a constitutive CMV promoter (pCMV-CLuc) or protein concentration. The pHRE-MP-GLuc HIF responsive construct has been previously described^58^. The pCMV-CLuc construct was from NEB (N0321S, pCMV-CLuc 2). Plasmid sequencing was performed by MWG Eurofins, Germany.

### Cell transfection with siRNA and transient REST overexpression construct

Transient transfection with siControl (sc-37007, SCB) and siREST (s11932, Life Technologies) were performed as previously described^42^. Transfections with siRNA to be used in luciferase assays were performed in 24 well plates, as described above. All other experiments were performed on 6 well plates unless otherwise stated. In a typical experiment 200 K cells were seeded on 6 well plates and allowed to grow until approximately 60% confluent, at this time cells were transfected with 2 μL of Lipo, 100 μL Optimem and a pre-optimized amount of overexpressing construct (100 ng) or siRNA (100 pmol). Cells were media changed to 2 mL of conditioned media on the day after transfection. In order to keep the same transfection times with the siRNA, in experiments were hypoxic exposures were longer than 16 hrs, cells were conditioned to hypoxia on different days and lysed on the same day. For RNA extraction experiments, siRNA was incubated for 48 hrs. For the preparation of whole cell protein extracts, siRNA was incubated for 72 hrs. For the experiments where the role of REST in the resolution of the HIF-1α response was investigated (Fig. 4A,B), HEK293 cells were stably transfected with a plasmid coding for a short-hairpin RNA (shRNA) targeting the coding sequence of REST mRNA (sc-38129-SH, Santa Cruz) or a control shRNA plasmid (sc-108060, Santa Cruz), and selected with puromycin, according to the manufacturer instructions. For experiments where REST was overexpressed using msREST-FLAG, 80 K cells were seeded on 12 well plates, and cells were transfected with 100 ng msREST-FLAG, 100 ng HREG and 50 ng pCMV-CLuc with 100 μL Optimem and 1 μL Lipo. The media was changed to fresh media on the day after transfection.

### qRT-PCR

cDNA was synthesized from 1 μg of RNA using MMLV (Promega), and amplified using the Prism 7900HT sequence detection system (Applied Biosystems, Foster City, CA) under default conditions. The mRNA relative expression was calculated by the ∆∆Ct method by normalizing the Ct of the samples to that of 18S rRNA (TaqMan Universal PCR Master Mix with the primer 18S rRNA-Euka, 4310893E, Life Technologies), followed by normalization to the control condition. The following qRT-PCR primers were used:

*HIF1A*, F: ACAAGTCACCACAGGACAG, R: CGACTTGATTTTCTCCCT

*HK2*, F: TCCCCTGCCACCAGACTA, R: CCAAGGGATTCAAGTCCA

*LDHA*, F: GAGGTTCACAAGCAGGTGGT, R: AGTGTTCCTTGCATTTTGGG

*SLC2A1*, F: GATTGGCTCCTTCTCTGTGG, R: AAACTGGGCAAGTCCTTTGA

*PFKFB3*, F: ACAGCTTTGAGGAGCATGTG, R: AAACATGAAAGGCTCCCG

PHD2, F: GCACGACACCGGGAAGTT, R: ACTGTAACGGGAAGCTGG

PHD3, F: ATCGACAGGCTGGTCCTCTA, R: ACAAGAATTGGGATGCCAAG

### Western blot

All reagents were from Sigma unless otherwise stated. Standard protocols were used as previously described^42^,^59^. Mouse β-actin, 1:10000, Sigma, A5441; Mouse HIF-1α,1:1000, BD Pharmingen, 610958; Rabbit HIF-2α, 1:1000, Novus Biologicals, NB100-122; Rabbit REST, 1:1000, Abcam, ab28018.

### Chromatin immunoprecipitation

ChIP assays where performed as previously described^60^. Briefly, HEK293 cells fully confluent on T175 flasks were conditioned to hypoxic media (1% oxygen) for the indicated time points. Cells were fixed with 2% formaldehyde in 10 mL fresh media for 10 min with agitation. Cells were removed from the hypoxic chamber and fixation was stopped with 125 mM glycine treatment for 5 min. The following mix was prepared: 1 uL of the purified DNA was used, 0.4 μL of 20 μM primers, 8 uL RNAse free water and 10 μL of Power SYBR^®^ Green (Applied Biosystems). The quantitative Real-Time PCR was performed using the 7900HT Fast Real-Time PCR System. Precipitated chromatin was normalized to input samples and the control IgG IP’s are shown as a negative control, as previously described^60^.

The following ChIP qRT-PCR primers were used:

HIF-RE1, F: AGAGGCTCGGAGCCGG, R: CGCTTCTCTCTAGTCTCACGAG

The following antibodies were used:

Rabbit CoREST, 5 μg, Millipore, 07–455; Rabbit REST, 2 μg, Millipore, 17–641; Rabbit mSin3A, 5 μg, SCB, sc-994; Rabbit IgG, 5 μg, Millipore, PP64B.

### Glucose and lactate quantification assays

For the lactate assays (Trinity Biotechnology Lactate Assay Kit, REF735-10), 1 μL of the supernatant or Lactate Standard Solution was incubated with 100 μL of the Lactate Reagent Solution (lactate oxidase, peroxidase, chromogenic precursor and a pH 7.2 buffer), incubated at room temperature for 10 min, absorbance at 540 nm was used to calculate the lactate concentration in the media, absorbance was corrected from blank. Lactate production relative to time zero was calculated and normalized to the protein concentration. For the glucose assays (Biovision, Glucose Assay Kit, REFK606-100) the media was diluted 1 in 10 in Assay Buffer, 1.5 μL of the diluted sample were mixed with 48 μL of the Assay Buffer, 1 μL of the Glucose Enzyme Mix and 1 μL of the Glucose probe. This was followed by incubation for 30 min at 37 °C, protected from light, and absorbance was measured at 570 nm to calculate glucose concentration in the media relative to a standard curve of a supplied glucose standard, absorbance was corrected from blank. Glucose consumption relative to time zero was calculated and normalized to the protein concentration. For HEK293 cells treated with siRNA, transfection was performed as described under the “Gaussia Luciferase Assays” section.

### Bioinformatics identification of the RE1 elements

ChIP-Seq data obtained by the ENCODE consortium across multiple cell lines and available at the UCSC genome browser (http://genome.ucsc.edu/) and the JASPAR database (http://jaspar.genereg.net/) of transcription factor binding sites, revealed the presence of a putative RE1 site on the HIF-1alpha promoter, located between 5207 and 5257 on the Homo sapiens *HIF1A* gene sequence on chromosome 14 (NCBI Reference Sequence: NG_029606.1). The Translation Start Site, referred to as ATG was determined by aligning the consensus coding sequence (CCDS) of HIF1A-001 (CCDS9753) to the HIF1A genomic DNA sequence. This corresponds to the uniprot entry (Q16665-1), the canonical HIF-1α isoform, with 826 amino acids (http://www.uniprot.org/uniprot/Q16665).

### Statistical analysis

All experiments were performed at least 3 independent times. All immunoblots shown are representative of biological replicates. Data is shown as mean ± SEM. Statistical significance was tested in Prism (Graphpad), using Student’s t test for the comparison of two data sets or ANOVA for more than two datasets. *p < 0.05, **p < 0.01 and ***p < 0.001.

## Additional Information

**How to cite this article**: Cavadas, M. A. S. *et al.* REST mediates resolution of HIF-dependent gene expression in prolonged hypoxia. *Sci. Rep.* **5**, 17851; doi: 10.1038/srep17851 (2015).

## Supplementary Material

Supplementary Information
