# Supplementary material for: REST mediates resolution of HIF-dependent gene expression in prolonged hypoxia
Source: Sci Rep. 2015 Dec 9;5:17851. doi: 10.1038/srep17851 (PMC4673454; doi:10.1038/srep17851)
Supplement: Supplementary Information [file srep17851-s1.pdf]

REST mediates resolution of HIF-dependent gene expression in prolonged hypoxia

Miguel A. S. Cavadas[1,2,3], Marion Mesnieres[2], Bianca Crifo[2], Mario C. Manresa[2], Andrew C. Selfridge[2], Carsten C. Scholz[1,2,4], Eoin P. Cummins[2], Alex Cheong[1,2,5,*], Cormac T. Taylor[1,2,*]

[1] Systems Biology Ireland, University College Dublin, Dublin 4, Ireland.

[2] Conway Institute of Biomolecular and Biomedical Research, School of Medicine and Medical Sciences, University College Dublin, Dublin 4, Ireland.

[3] Instituto Gulbenkian de Ciencia, Rua da Quinta Grande, 2780-156 Oeiras, Portugal.

[4] Institute of Physiology and Zurich Centre for Integrative Human Physiology, University of Zurich, Zurich, Switzerland.

[5] Life and Health Sciences, Aston University, Birmingham, B4 7ET, UK.

[*] These authors contributed equally to this work

Supplementary Figures

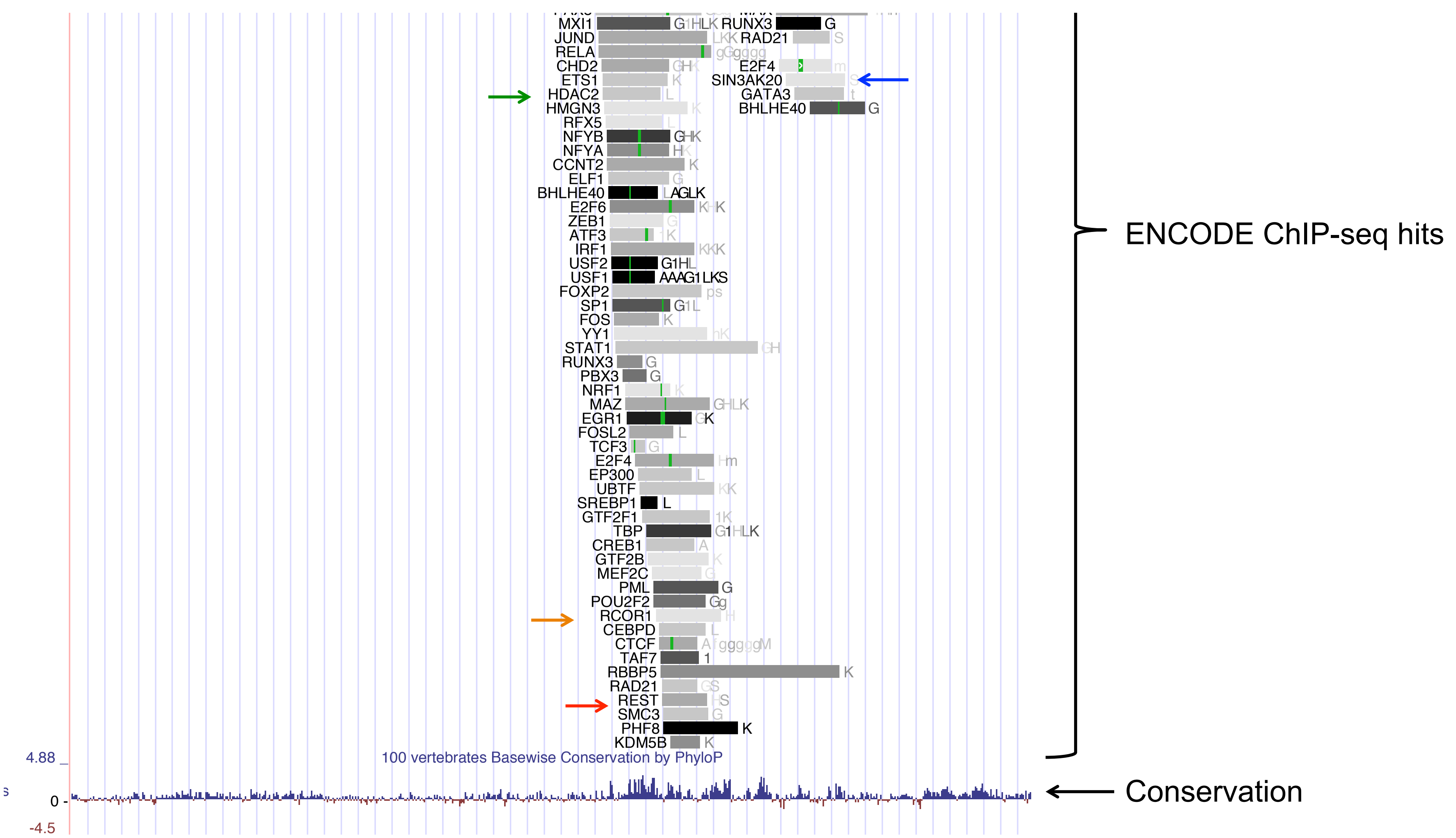
ENCODE ChIP-seq hits
Conservation
100 vertebrates Basewise Conservation by PhyloP
4.88
0
-4.5
MXI1
JUND
RELA
CHD2
ETS1
HDAC2
HMGN3
RFX5
NFYB
NFYA
CCNT2
ELF1
BHLHE40
E2F6
ZEB1
ATF3
IRF1
USF2
USF1
FOXP2
SP1
FOS
YY1
STAT1
RUNX3
PBX3
NRF1
MAZ
EGR1
FOSL2
TCF3
E2F4
EP300
UBTF
SREBP1
GTF2F1
TBP
CREB1
GTF2B
MEF2C
PML
POU2F2
RCOR1
CEBPD
CTCF
TAF7
RBBP5
RAD21
REST
SMC3
PHF8
KDM5B
RUNX3
RAD21
E2F4
SIN3AK20
GATA3
BHLHE40

Figure S1. ChIP-seq hits from the ENCODE datasets on the HIF1A genomic locus -4000 to +2000 bp relative to the ATG. The *HIF1A* gene with mammalian conservation is depicted in relation to its position on chromosome 14 q23.2. The human *HIF1A* promoter region -4000 bp upstream to +2000 bp downstream of the translation start site (ATG) was used to screen for the presence of REST-repressor complex components on the ENCODE ChIP-seq datasets. This is a highly conserved region surrounding EXON1. The full list of transcription factors found to be associated on this region of the HIF1A gene is shown. ChIP-seq hits found for the REST co-repressor complex components: REST, mSin3A, CoREST (RCOR1) and HDAC2 on the HIF1A gene promoter are colour coded and highlighted by a horizontal arrow bar. Conservation is depicted by vertical blue bars, below the ChIP-seq hits (horizontal grey-scale bars). More details on the bioinformatics analysis can be found on the Materials and Methods section.

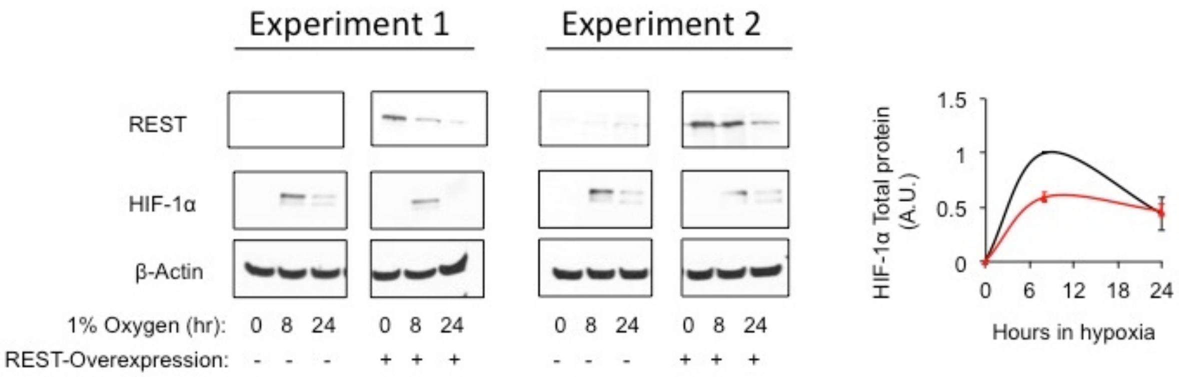

Figure S2. REST-overexpression supresses HIF-1α protein levels. HEK293 cells where exposed to hypoxia for the indicated time points, before cell lysis. Western blots where performed with the indicated antibodies. Blots from 2 independent experiments are show, together with the densitometry of HIF-1a protein levels from 3 independent experiments. REST was overexpressed in HEK293 cells as described for the luciferase assay in Figure 4D.

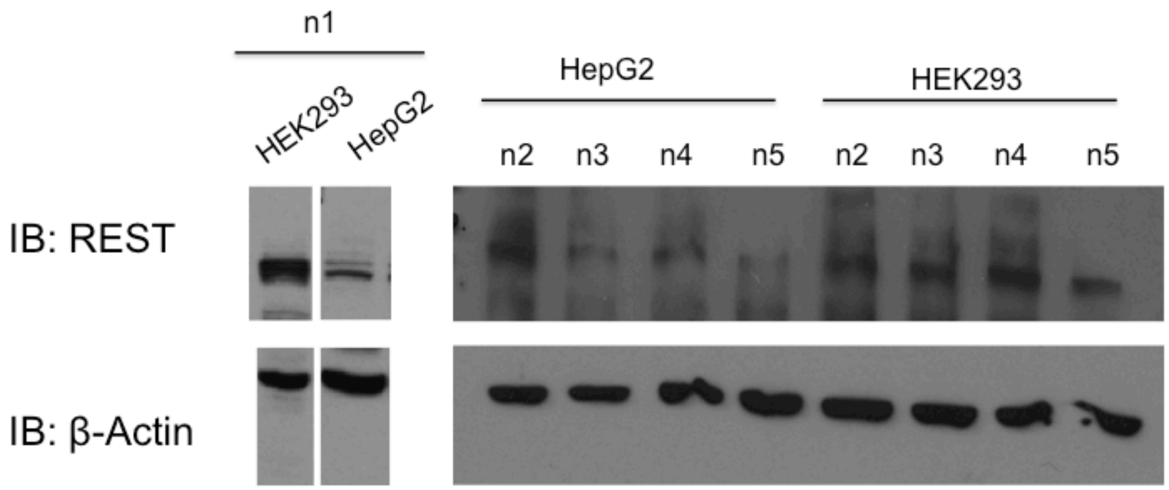

Figure S3. REST expression in HEPG2 and HEK293 cells. Immuno-Blots (IB) were performed with the indicated antibodies, on extracts collected from 5 independent HepG2 and HEK293 cultures. REST levels are higher in HEK293 cells, across 4 out of 5 independent experiments (n1 to n5).
